# Supplementary figures and images for: Single-nucleus RNA sequencing reveals cell type-specific responses to heat stress in bovine mammary gland
Source: J Anim Sci Biotechnol. 2026 Jul 16;17:148. doi: 10.1186/s40104-026-01468-x (PMC13374132; doi:10.1186/s40104-026-01468-x)

**A**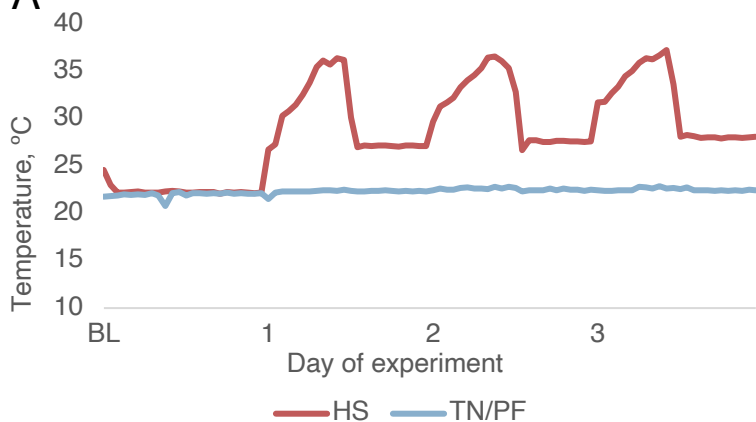**B**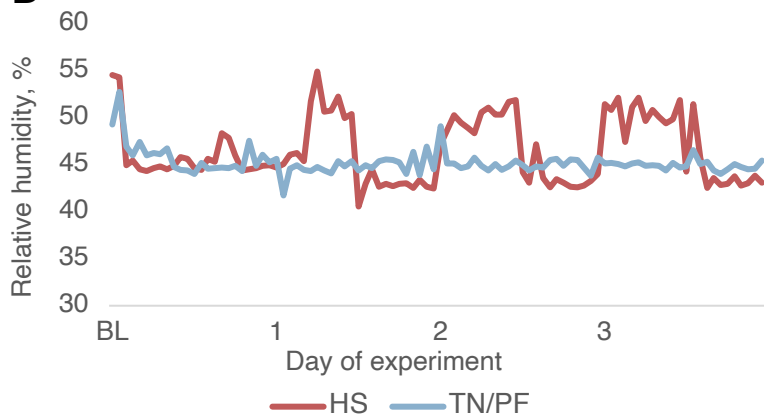**C**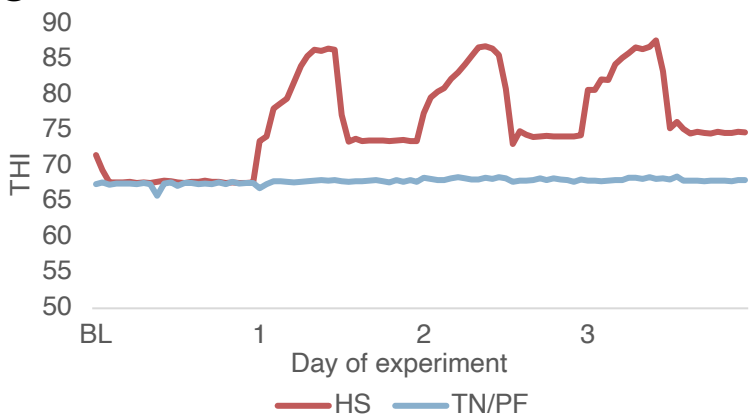

Supplement: Supplementary file 1 — Additional file 1: Fig. S1. Environmental conditions in respiration chambers during the experimental period. Data were averaged hourly throughout the experiment forambient temperature,relative humidity, andtemperature-humidity index. BL, baseline values during acclimation period; HS, heat stress; TN, thermoneutrality; PF, thermoneutrality and pair-fed. [file 40104_2026_1468_MOESM1_ESM.pdf]

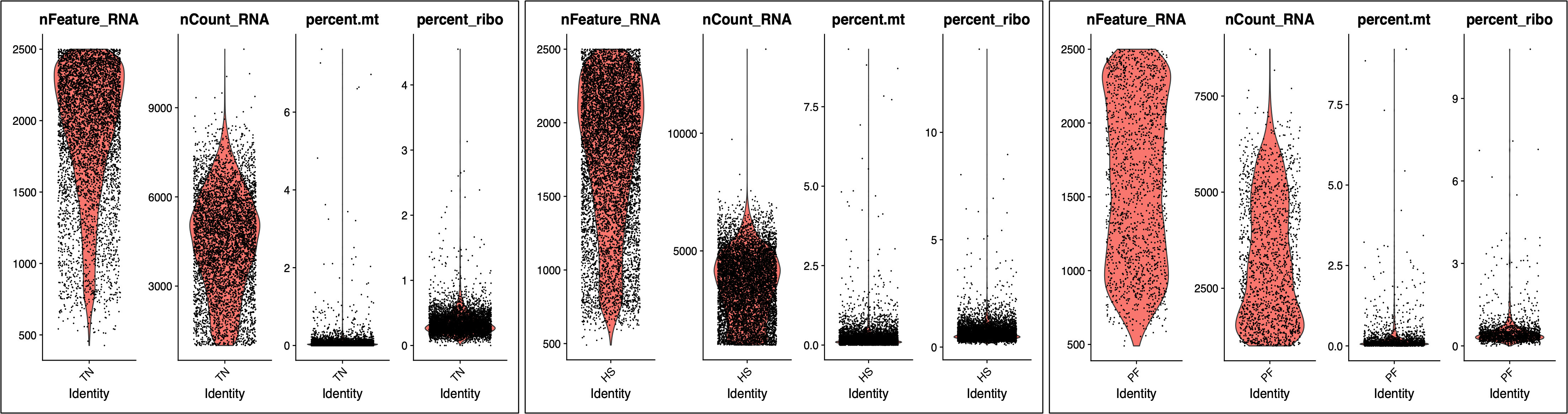

Supplement: Supplementary file 4 — Additional file 4: Fig. S2. Quality control metrics for snRNA-seq data. Quality control of snRNA-seq data forTN,HS, andPF samples. [file 40104_2026_1468_MOESM4_ESM.png]

**ELF5**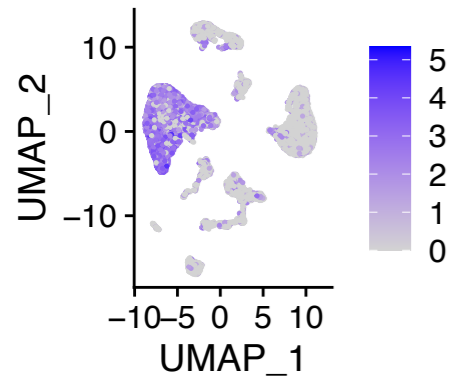**GLYCAM1**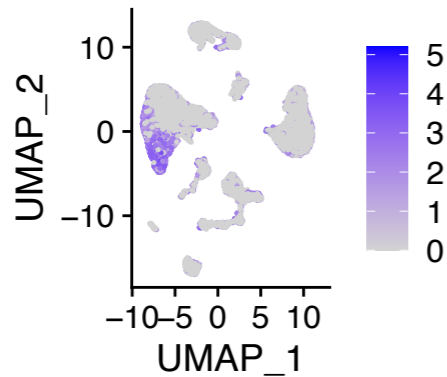**COL1A1**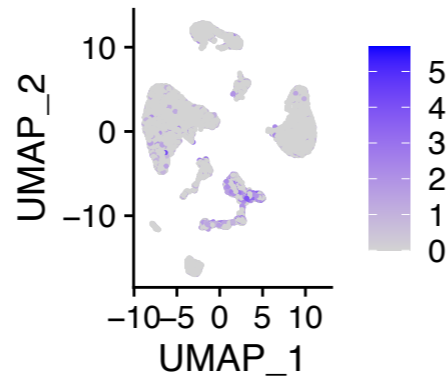**NOTCH3**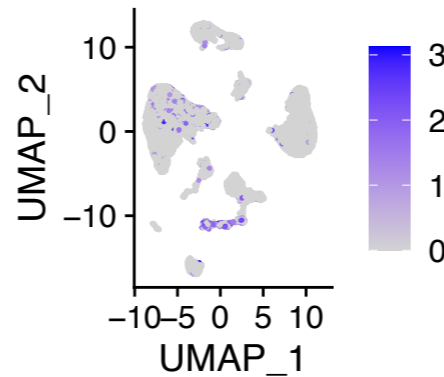**CD3E**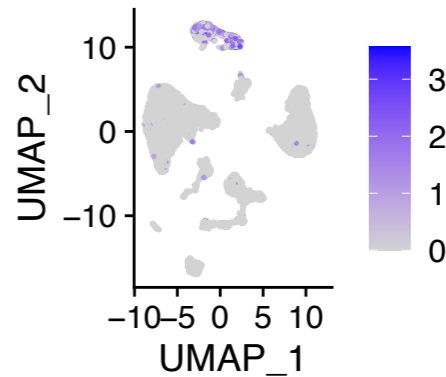**TXK**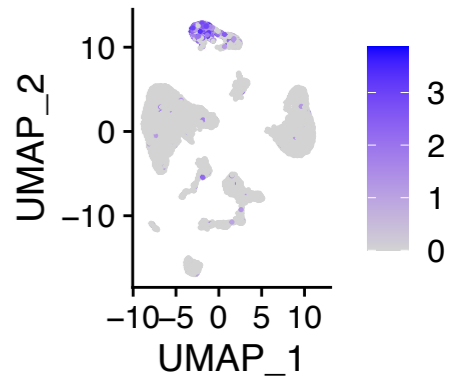**PRLR**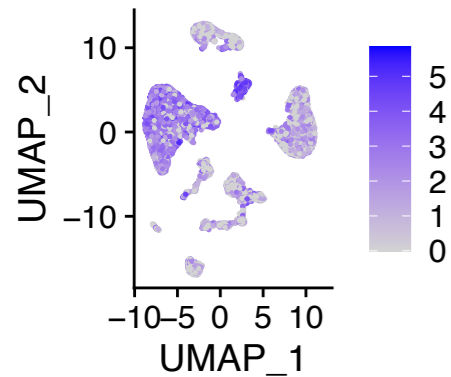**ESR1**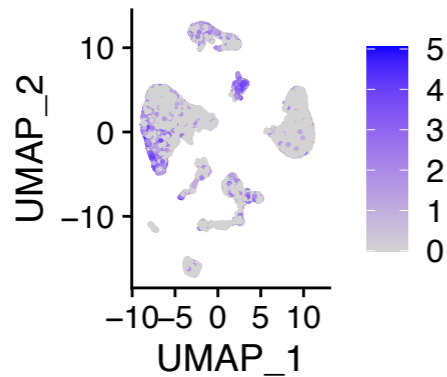**PECAM1**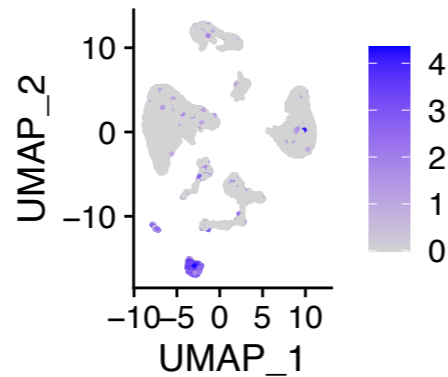**FLT4**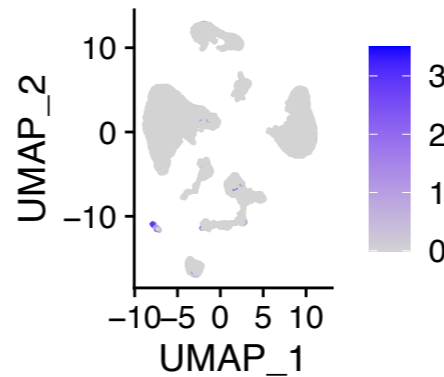**CD27**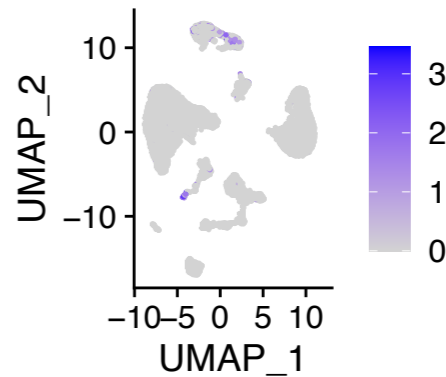**CD86**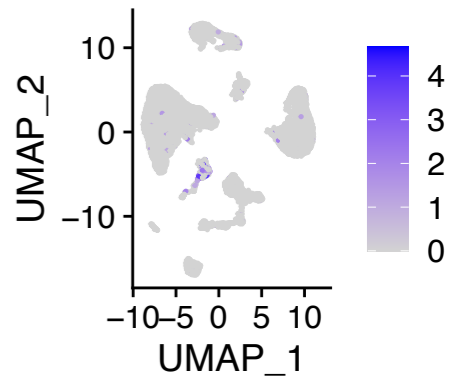

Supplement: Supplementary file 5 — Additional file 5: Fig. S3. Feature plots of marker gene expression. Feature plots showing expression of marker genes for identified clusters. [file 40104_2026_1468_MOESM5_ESM.pdf]

A

Cell type proportion

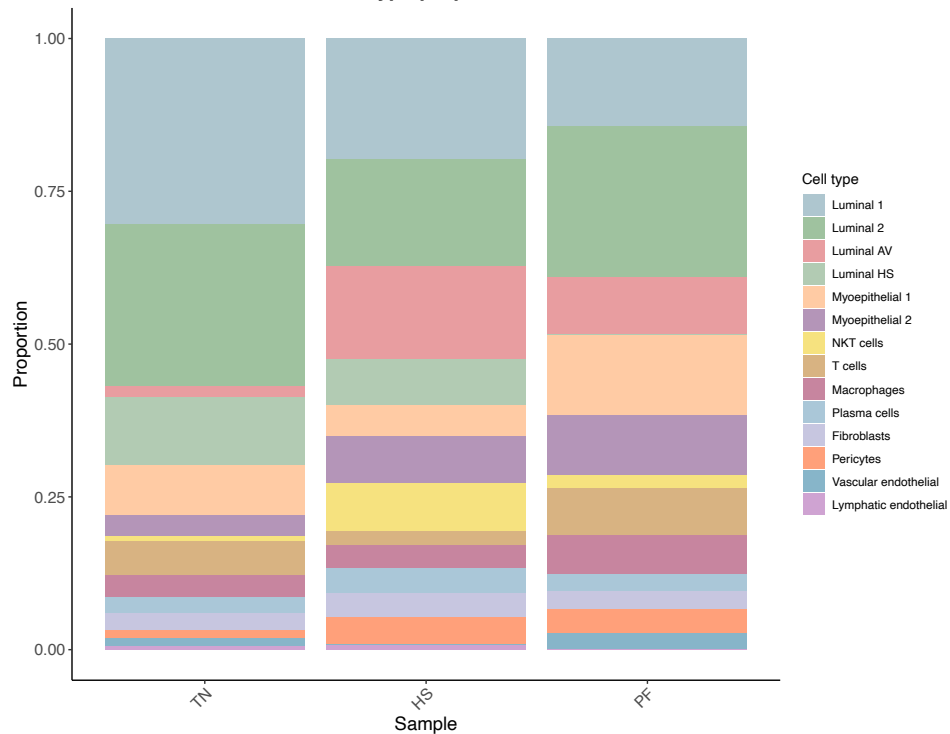

B

Cell number per sample

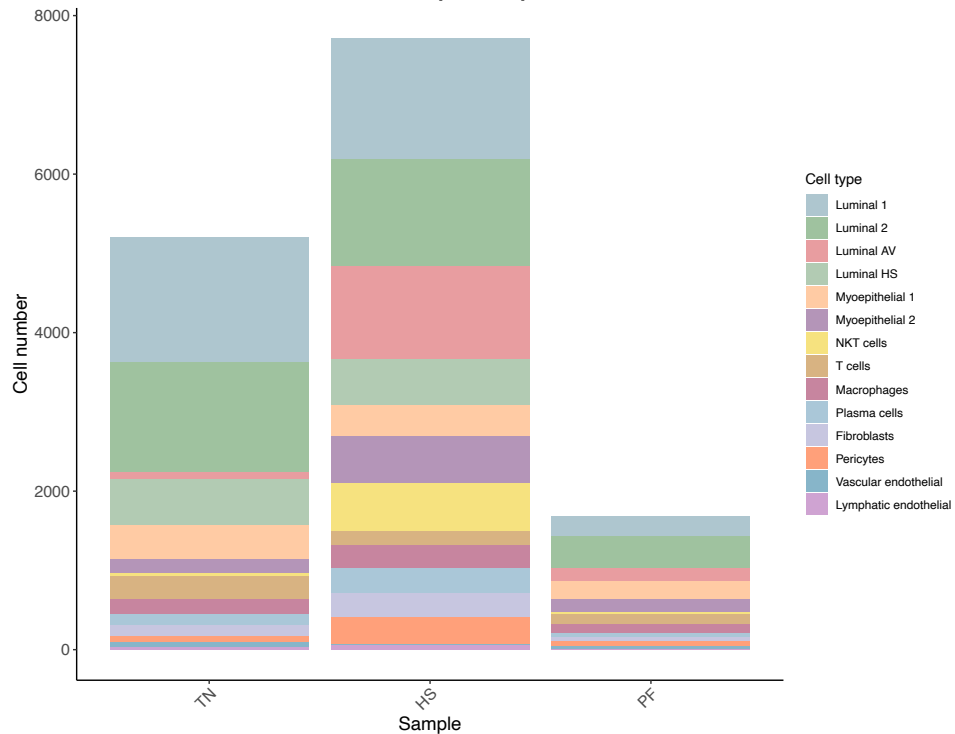

Supplement: Supplementary file 6 — Additional file 6: Fig. S4. Cell proportion and counts across samples. A. Proportion of each cell cluster in individual samples. B. Total cell counts in TN, HS, and PF samples. [file 40104_2026_1468_MOESM6_ESM.pdf]

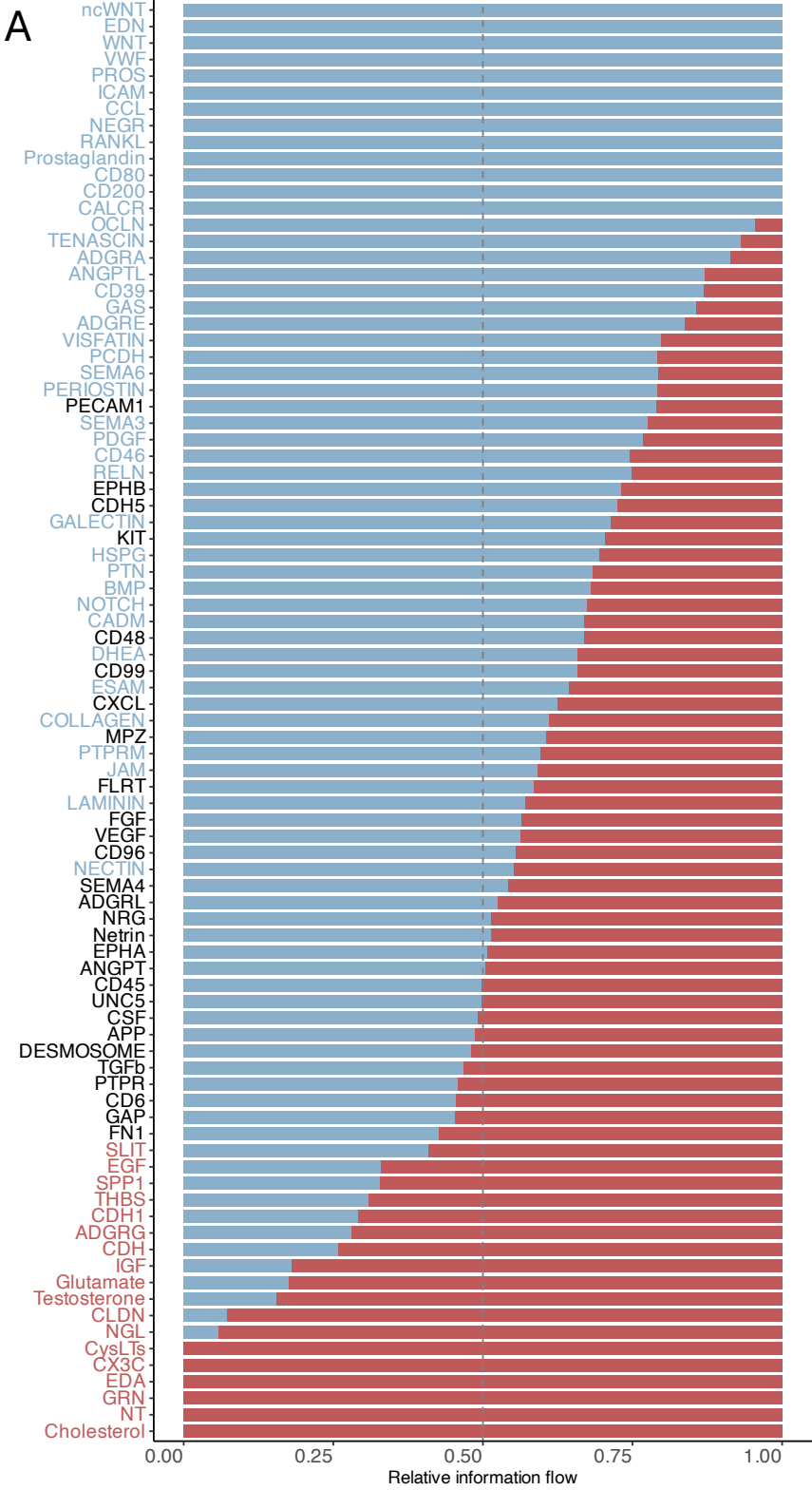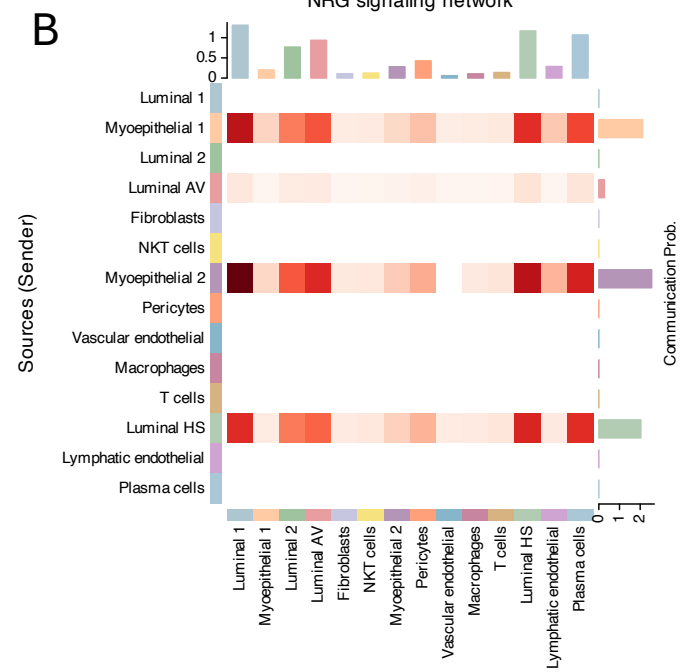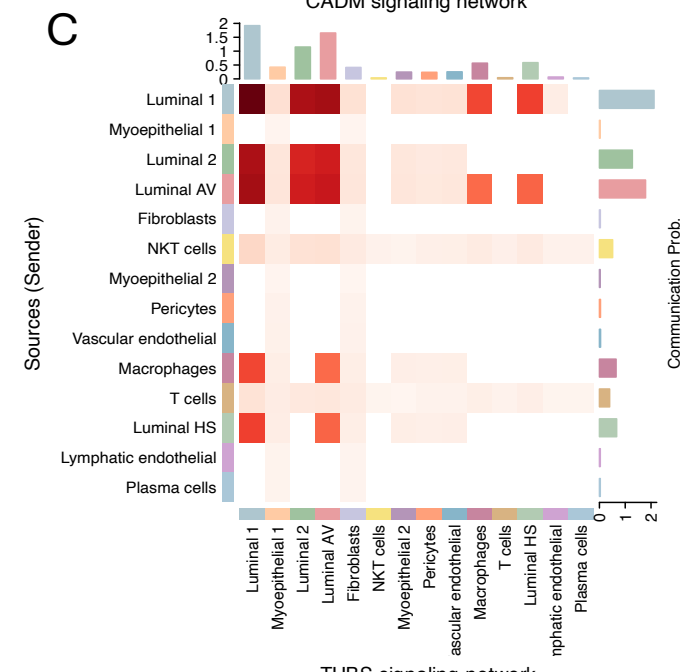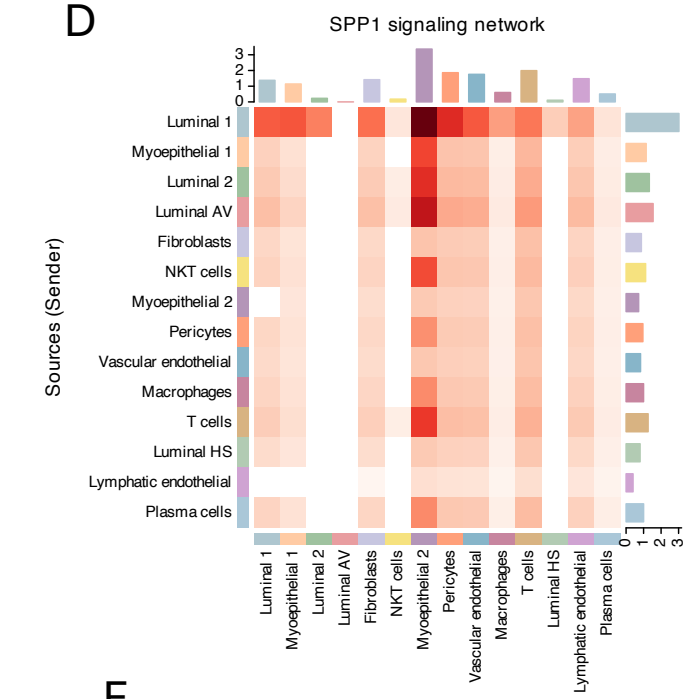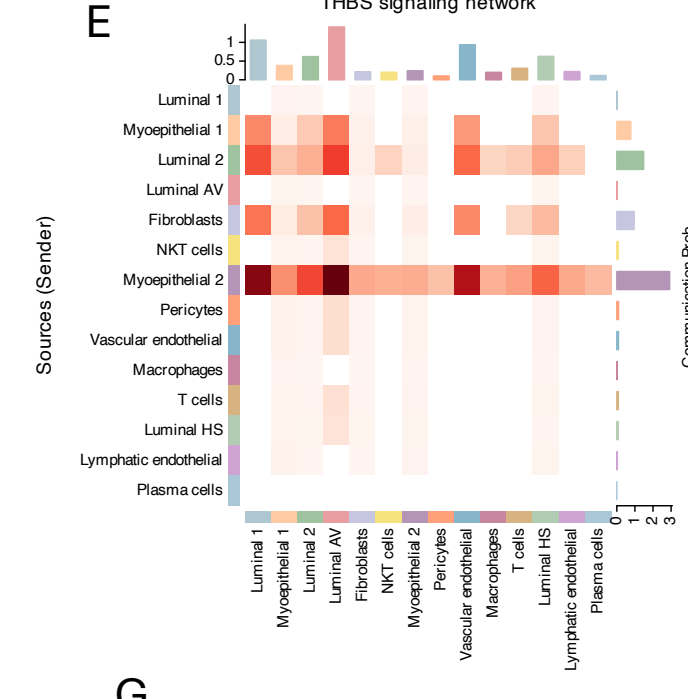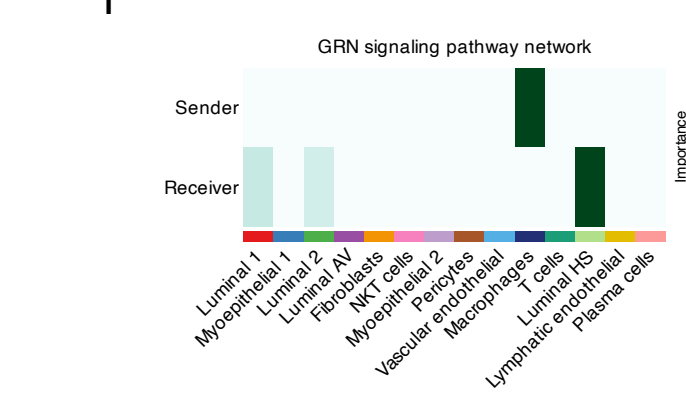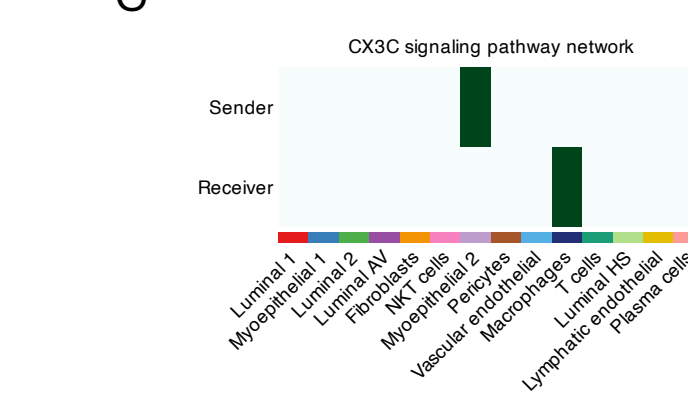

Supplement: Supplementary file 9 — Additional file 9: Fig. S5. Signaling pathways enriched in TN or HS conditions. A. Signaling pathways enriched in TN or HS conditions. B–E. Intercellular communication networks for selected signaling pathways among identified clusters. F–G. Selected signaling pathway network detected under HS condition. NRG, neuregulin; CADM, cell adhesion molecules; SPP1, secreted phosphoprotein 1; THBS, thrombospondin. GRN, granulin; CX3C, C-X3-C motif chemokine. [file 40104_2026_1468_MOESM9_ESM.pdf]

A

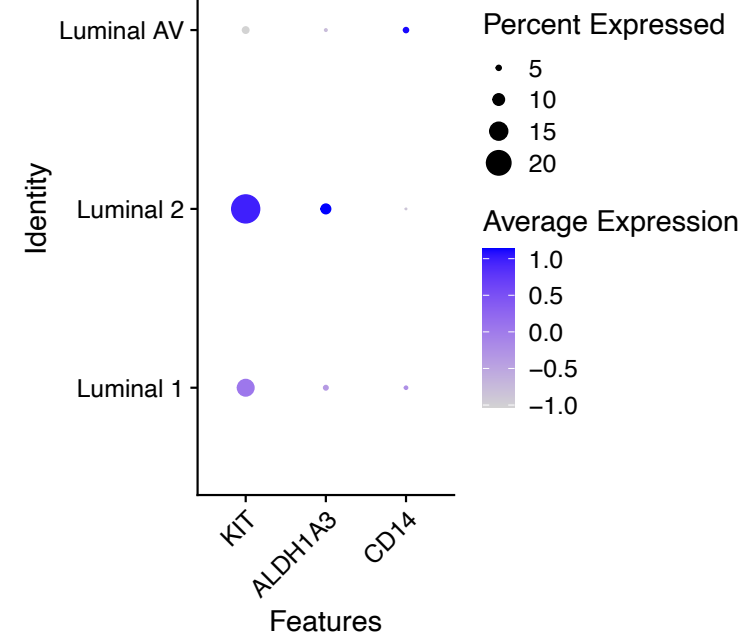

B

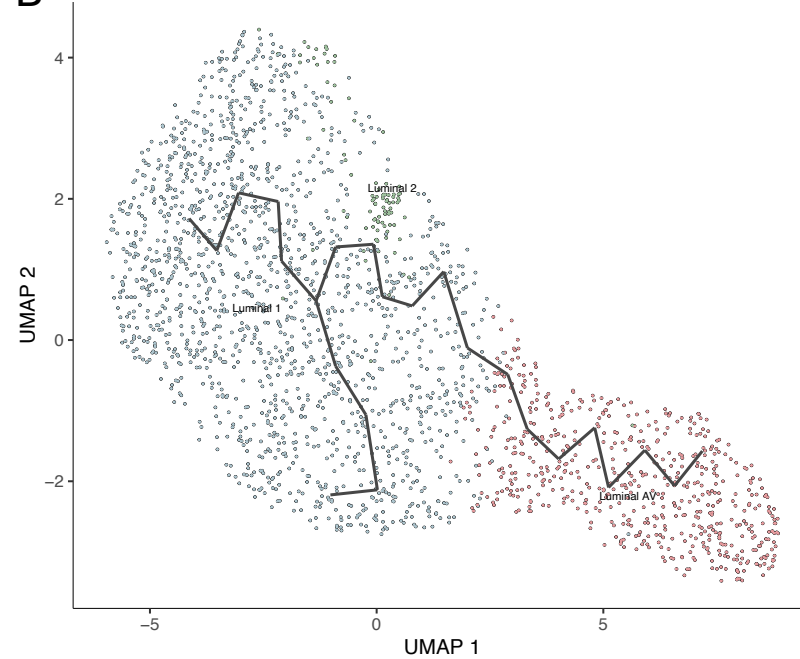

C

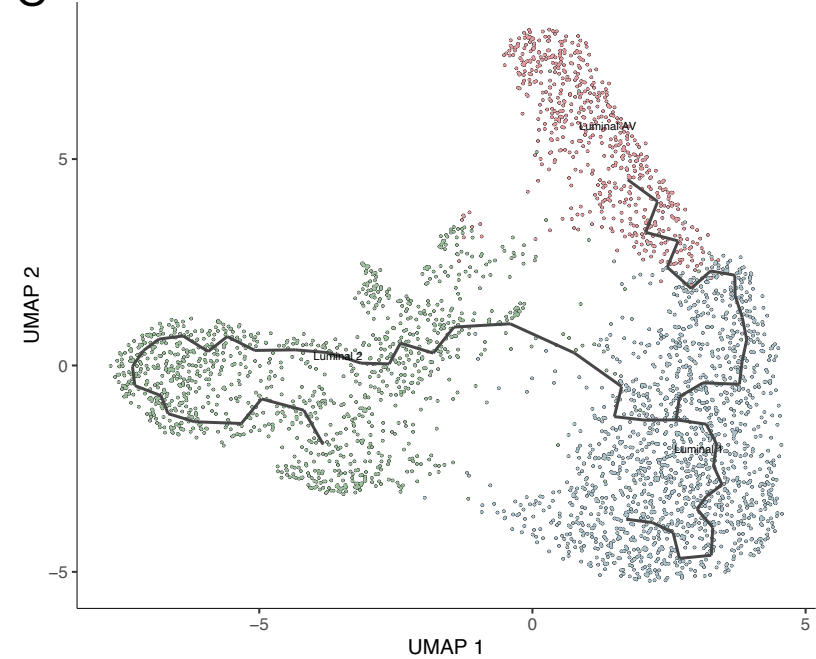

Supplement: Supplementary file 10 — Additional file 10: Fig. S6. Trajectory analysis of luminal clusters. A. Dot plot showing expression of luminal progenitor marker genes in Luminal 1, Luminal 2, and Luminal AV clusters. B–C. Trajectory maps of luminal cells with clusters labeled inTN condition andHS condition. KIT, KIT proto-oncogene, receptor tyrosine kinase; ALDH1A3, aldehyde dehydrogenase 1 family member A3; CD14, monocyte differentiation antigen CD14. [file 40104_2026_1468_MOESM10_ESM.pdf]
