# Supplementary material for: Single-nucleus RNA sequencing reveals cell type-specific responses to heat stress in bovine mammary gland
Source: J Anim Sci Biotechnol. 2026 Jul 16;17:148. doi: 10.1186/s40104-026-01468-x (PMC13374132; doi:10.1186/s40104-026-01468-x)
Supplement: Supplementary file 2 — Additional file 2: Table S1. Effects of heat stress on clinical assessments of lactating multiparous Holstein cows. Rectal temperature, skin temperature, respiration rate, and heart rate were measured three times dailyin cows maintained under TN, HS, or PF conditions. [file 40104_2026_1468_MOESM2_ESM.docx]

**Additional file 2: Table S1** Effects of heat stress on clinical assessments of lactating multiparous Holstein cows

| **Variable, unit** | **Treatment^1^** | | |  | ***P*-values^2^** | | | |
| --- | --- | --- | --- | --- | --- | --- | --- | --- |
|  | **TN** | **PF** | **HS** |  | **Treatment** | **HS vs. TN** | **HS vs. PF** |  |
| Rectal temperature, °C |  |  |  |  | <0.01 | <0.01 | <0.01 |  |
| 0700 h | 38.4 | 38.2 | 39.2 |  |  |  |  |  |
| 1200 h | 38.4 | 38.2 | 40.0 |  |  |  |  |  |
| 1900 h | 38.6 | 38.1 | 40.0 |  |  |  |  |  |
| Skin temperature, °C |  |  |  |  | <0.01 | <0.01 | <0.01 |  |
| 0700 h | 35.5 | 34.6 | 35.3 |  |  |  |  |  |
| 1200 h | 34.8 | 33.4 | 37.2 |  |  |  |  |  |
| 1900 h | 35.1 | 34.0 | 37.2 |  |  |  |  |  |
| Respiration rate, resp/min |  |  |  |  | <0.01 | <0.01 | <0.01 |  |
| 0700 h | 54.5 | 49.2 | 86.6 |  |  |  |  |  |
| 1200 h | 60.5 | 44.9 | 93.6 |  |  |  |  |  |
| 1900 h | 58.7 | 49.7 | 95.1 |  |  |  |  |  |
| Heart rate, beats/min |  |  |  |  | <0.01 | <0.01 | <0.01 |  |
| 0700 h | 71.3 | 76.7 | 81.8 |  |  |  |  |  |
| 1200 h | 72.2 | 69.3 | 83.1 |  |  |  |  |  |
| 1900 h | 75.4 | 71.1 | 83.6 |  |  |  |  |  |

^1^Nine pregnant multiparous and lactating Holstein cows were randomly assigned to one of three environmental treatments: thermoneutrality (TN; *n* = 3, THI = 68), heat-stress (HS; *n* = 3, THI = 74–86), thermoneutrality but pair-fed to HS (PF, *n* = 2, THI = 68)

^2^Statistical significance was considered when *P* ≤ 0.05. Trend towards significance was considered when 0.05 < *P* ≤ 0.15
