# Supplementary material for: Single-nucleus RNA sequencing reveals cell type-specific responses to heat stress in bovine mammary gland
Source: J Anim Sci Biotechnol. 2026 Jul 16;17:148. doi: 10.1186/s40104-026-01468-x (PMC13374132; doi:10.1186/s40104-026-01468-x)
Supplement: Supplementary file 3 — Additional file 3: Table S2. Summary of sequencing and quality control metrics for snRNA-seq libraries. Summary statistics of sequencing depth and quality control metrics for nuclei from TN, HS, and PF samples. [file 40104_2026_1468_MOESM3_ESM.docx]

| Treatment^1^ | Cell ranger | | Quality control | |
| --- | --- | --- | --- | --- |
|  | **Estimated cell number** | **Mean reads per cell** | **Median nCount_RNA per cell** | **Number of cells analyzed** |
| TN | 9,009 | 55,540 | 4,468 | 5,201 |
| HS | 12,929 | 38,073 | 3,534 | 7,711 |
| PF | 4,521 | 126,837 | 3,239 | 1,619 |

**Additional file 3: Table S2** Summary of sequencing and quality control metrics for snRNA-seq libraries

^1^Nine pregnant multiparous and lactating Holstein cows were randomly assigned to one of three environmental treatments: thermoneutrality (TN; *n* = 3, THI = 68), heat-stress (HS; *n* = 3, THI = 74–86), thermoneutrality but pair-fed to HS (PF, *n* = 2, THI = 68). Mammary gland biopsies from animals in each group were pooled and subject to snRNA-seq
