# Supplementary material for: Single-nucleus RNA sequencing reveals cell type-specific responses to heat stress in bovine mammary gland
Source: J Anim Sci Biotechnol. 2026 Jul 16;17:148. doi: 10.1186/s40104-026-01468-x (PMC13374132; doi:10.1186/s40104-026-01468-x)
Supplement: Supplementary file 7 — Additional file 7: Table S3. Cell counts per cluster and condition. Number of cells in each cluster in TN, HS, and PF conditions. [file 40104_2026_1468_MOESM7_ESM.docx]

| Cell cluster | Treatment^1^ | | |
| --- | --- | --- | --- |
|  | **TN** | **HS** | **PF** |
| Luminal 1 | 1,583 | 1,517 | 242 |
| Luminal 2 | 1,376 | 1,358 | 413 |
| Luminal AV | 94 | 1,165 | 158 |
| Luminal HS | 579 | 588 | 2 |
| Myoepithelial 1 | 426 | 395 | 220 |
| Myoepithelial 2 | 180 | 581 | 164 |
| NKT cells | 39 | 618 | 36 |
| T cells | 289 | 179 | 128 |
| Macrophages | 190 | 281 | 107 |
| Plasma cells | 135 | 318 | 49 |
| Fibroblasts | 142 | 304 | 50 |
| Pericytes | 71 | 338 | 66 |
| Vascular endothelial | 70 | 11 | 42 |
| Lymphatic endothelial | 27 | 58 | 2 |
| Total | 5,201 | 7,711 | 1,679 |

**Additional file 7: Table S3** Cell counts per cluster and condition

^1^Nine pregnant multiparous and lactating Holstein cows were randomly assigned to one of three environmental treatments: thermoneutrality (TN; *n* = 3, THI = 68), heat-stress (HS; *n* = 3, THI = 74–86), thermoneutrality but pair-fed to HS (PF, *n* = 2, THI = 68). Mammary gland biopsies from animals in each group were pooled and subject to snRNA-seq
